# Insights to Resistive Pulse Sensing of Microparticle and Biological Cells on Microfluidic Chip

**DOI:** 10.3390/bios15080496

**Published:** 2025-08-01

**Authors:** Yiming Yao, Kai Zhao, Haoxin Jia, Zhengxing Wei, Yiyang Huo, Yi Zhang, Kaihuan Zhang

**Affiliations:** 1 Liaoning Key Laboratory of Marine Sensing and Intelligent Detection, Department of Information Science and Technology, Dalian Maritime University, Dalian 116026, China; 2 2020 X-Lab, Shanghai Institute of Microsystem and Information Technology, Chinese Academy of Sciences, Shanghai 200050, China; 3 Houston International Institute, Dalian Maritime University, Dalian 116026, China

**Keywords:** resistive pulse sensing, microfluidics, tunable detection, micro-target

## Abstract

Since the initial use of biological ion channels to detect single-stranded genomic base pair differences, label-free and highly sensitive resistive pulse sensing (RPS) with nanopores has made remarkable progress in single-molecule analysis. By monitoring transient ionic current disruptions caused by molecules translocating through a nanopore, this technology offers detailed insights into the structure, charge, and dynamics of the analytes. In this work, the RPS platforms based on biological, solid-state, and other sensing pores, detailing their latest research progress and applications, are reviewed. Their core capability is the high-precision characterization of tiny particles, ions, and nucleotides, which are widely used in biomedicine, clinical diagnosis, and environmental monitoring. However, current RPS methods involve bottlenecks, including limited sensitivity (weak signals from sub-nanometer targets with low SNR), complex sample interference (high false positives from ionic strength, etc.), and field consistency (solid-state channel drift, short-lived bio-pores failing POCT needs). To overcome this, bio-solid-state fusion channels, in-well reactors, deep learning models, and transfer learning provide various options. Evolving into an intelligent sensing ecosystem, RPS is expected to become a universal platform linking basic research, precision medicine, and on-site rapid detection.

## 1. Introduction

Resistance Pulse Sensing (RPS), commonly referred to as the Coulter principle, is a sensing technique that relies on resistance variations to detect particles or molecules [1,2]. Following its initial development by Coulter for cell enumeration, RPS has established itself as a central technique for particle detection and characterization owing to its high sensitivity [3,4], single-particle readout capability [5,6,7], and broad applicability [8,9,10]. Kuzmanovic et al. reignited interest in RPS by using biological α-hemolysin pores to detect single-stranded DNA, demonstrating the ability to distinguish subtle size differences among the four DNA base pairs. This breakthrough has sparked significant interest in the use of nanopores for DNA sequencing [11,12]. With advances in nanofabrication technology, the design and application of RPS sensors have expanded significantly, evolving from traditional Coulter counters to modern microfluidic and nanofluidic sensors [13,14]. These advanced sensors are now widely used to detect single molecules, DNA, viruses, nanoparticles, and other nanoscale entities [15,16,17,18,19,20].

In recent years, the RPS technique has shown remarkable progress in microfluidic and nanofluidic applications [21,22]. Through nanofabrication techniques, researchers can precisely control and customize the size, surface chemistry, and mechanical stability of nanopores, thereby significantly enhancing the sensitivity and throughput of sensors [23,24]. Additionally, innovative designs of microfluidic and elastic pore devices have further improved the characterization capabilities of nanoscale objects, such as serial, parallel, and tunable-sized pores [25,26]. These technological advancements allow RPS sensors to detect and characterize the size, shape, charge, and concentration of nanoscale entities, such as proteins, viruses, exosomes, and liposomes, providing powerful tools for applications in biomedical research, clinical diagnostics, and environmental monitoring [27,28,29].

While traditional flow cytometers have achieved great success in cell detection and counting, their limitations, such as bulky size, high cost, and inability to handle small sample volumes [30,31,32], have driven the development of portable and low-cost RPS sensors. Microfluidic and nanofluidic RPS sensors not only retain the high sensitivity and accuracy of traditional RPS technology but also enable the high-precision characterization of nanoparticles, DNA, viruses, and antigens [33,34] through the integration of nanopore design and microfluidic technology. For instance, resistance pulse signals can be used to infer the particle shape, charge, and conductivity, offering new methods for studying the physicochemical properties of particles [35,36,37].

**Figure 1:**
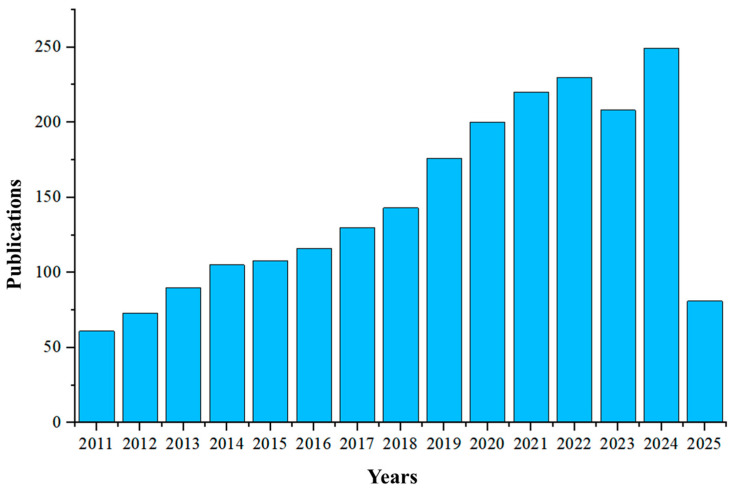
The number of papers published in the RPS-related field over time. The data were obtained from the Web of Science by searching the keyword “resistance pulse sensing”.

This work aims to examine the latest advancements in RPS methods, including the fundamental principles of the PRS technique, the materials of the microfluidic chip, fabrication processes, and corresponding applications. The publications related to RPS over the past 10 years are shown in Figure 1. Especially, to accurately size microparticles and biological cells in electrolyte solutions, a three-way classification framework of biological, solid-state, and other inductive pores is reviewed. Lastly, future research directions and challenges within this domain are explored and summarized.

## 2. Theory of RPS

**Figure 2:**
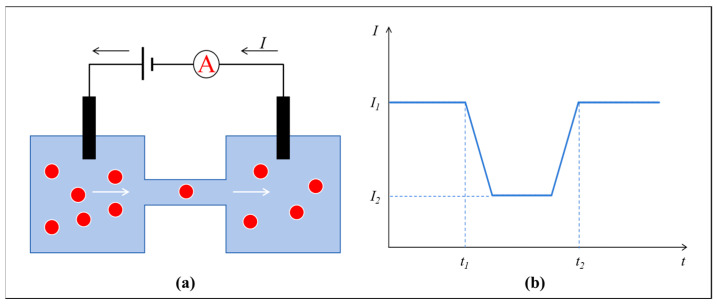
Schematic illustration of the RPS working principle in a microfluidic chip. (**a**) Schematic diagram, (**b**) Current change diagram.

RPS technology functions by measuring the variations in the electrical resistance induced by particles traversing a sensing orifice. This principle has been applied to microfluidic and nanofluidic systems, thereby facilitating accurate particle enumeration and sizing [38]. Figure 2 illustrates the working principle of the RPS sensing technique.

The RPS method employs the principle of the Coulter counter in a microfluidic chip to determine the diameter and number of particles. Generally, an electrolyte solution occupies the main channel and the sensing orifice. An electric field is imposed across the sensing orifice, which has a diameter that is significantly smaller than that of the main channel. As a particle traverses the sensing orifice, it displaces an electrolyte volume, leading to transient resistance variation. Then, a single pulse is induced for each individual particle, the magnitude of which scales linearly with the volume ratio between the particle and sensing orifice. If the particle is less conductive (or non-conductive) than the electrolyte, this displacement reduces the overall conductivity of the current path through the orifice, i.e., effectively increasing the resistance, and hence the transient resistance spike. In contrast, if a particle is more conductive than the electrolyte, displacement enhances the local conductivity, leading to a transient decrease in resistance. This resistance variation is detected as a voltage or current pulse and processed using amplification circuits, data acquisition devices, and corresponding reading software. An individual target generates a single signal pulse, with the signal amplitude proportional to the particle-to-orifice volume ratio. This correlation enables simultaneous particle size determination and enumeration [9]. The fundamental equation governing this principle is derived from Ohm’s law and charge conservation. The resistance variation (∆R) induced by the target entering the sensing channel can be expressed as

(1) $$∆R=\frac{\rho \cdot L}{A_{eff}}$$

where ρ is the resistivity of the electrolyte solution, L is the length of the sensing orifice, and $A_{eff}$ is the effective cross-sectional region of the sensing orifice. When the particle of volume $V_{P}$ enters the orifice, the effective area is reduced, leading to an increase in resistance [39]. The detection sensitivity, i.e., the resistance variation, is mainly determined by the volume ratio between the particles and the gate [40].

(2) $$∆R=\frac{{4\rho d}^{3}}{{\pi D}^{4}}\left[1+\frac{{2d}^{3}}{3D^{2}L}+{\left(\frac{{2d}^{3}}{3D^{2}L}\right)}^{2}+\ldots \right]\;\left(d\ll D\right)$$

A critical aspect of the RPS is establishing the relationship between the measured signal magnitude and particle size. This relationship is determined through the calculation of resistance variation induced by the micro-target entering the sensing orifice (∆R) [36]. Based on the assumption that the spherical particles flow along the centerline of a cylindrical channel, the relative resistance change is expressed as

(3) $$\frac{∆R}{R}=\frac{d^{3}}{d^{3}+D^{3}}$$

where d and D are the particle diameter and sensing orifice diameter, respectively, indicating that the resistance change is highly dependent on the particle-to-orifice size ratio [41]. The relative resistance variations induced by spherical particles of different diameters were compiled. Larger particles create greater resistance changes, making them easier to detect than smaller particles. However, smaller particles (near the sensor pore size) can also be detected with sufficient sensitivity if the system is properly calibrated [13]. In cases where the particle’s diameter is considerably smaller than the orifice’s diameter, the relative change in resistance is given by

(4) $$\frac{∆R}{R}=\frac{d^{3}}{D^{2}L}\left[\frac{D^{2}}{{2L}^{2}}+\frac{1}{\sqrt{1+{\left(D/L\right)}^{2}}}\right]F\left(\frac{d^{3}}{D^{3}}\right)$$

where L is its length. When the particle’s diameter is medium relative to the pore’s diameter, the relative resistance change is expressed as

(5) $$\frac{∆R}{R}=\frac{d^{3}}{D^{2}L}\cdot \frac{1}{1-0.8{\left(d/D\right)}^{3}}$$

For larger particles, the relative resistance change is expressed as

(6) $$\frac{∆R}{R}=\frac{D}{L}\left[\frac{arcsin\left(d/D\right)}{\sqrt{1-{\left(d/D\right)}^{2}}}-\frac{d}{D}\right]$$

A general expression for the resistance changes due to a particle in the pore is given by

(7) $$\frac{∆R}{R}=f\cdot v\cdot V_{S}\cdot \frac{d}{D}$$

where f is the particle shape factor, v is the particle volume, $V_{S}$ is the sensing volume, d is the particle diameter, for spherical particles, f is 3/2 as per Maxwell’s theory. The shape factor f varies for non-spherical particles, such as oblate and prolate spheroids, depending on their orientation and aspect ratio. An empirical correction factor $S\left(d/D\right)$ accounts for the deviation from the linear relationship between the volume of micro-target and the magnitude of the sensing pulse, resulting from the bulging of the electric field when the particle diameter is close to the pore diameter [42]

(8) $$S\left(d/D\right)=\frac{1}{1-0.8{\left(d/D\right)}^{3}}$$

The frequency of the resistive pulses, known as the blockade frequency, determines the particle concentration, which correlates with the particle dispersion concentration in the electrolyte solution via the Nernst-Planck equation

(9) $$J=\frac{\pi v_{s}D^{2}}{4C}$$

where J is the current density, $v_{s}$ is the particle velocity, C and D illustrate the particle concentration and the pore diameter. The particle velocity $v_{s}$ can be influenced by various factors, including electrically driven flow, diffusion, and external pressure [3]. The duration of the resistive pulse $\left(∆t\right)$ is affected by multiple factors, including the particle’s velocity due to electrophoretic motion ($v_{\mu }$), the velocity due to electro-osmotic flow ($v_{p}$), and the velocity due to external pressure ($v_{\varepsilon }$)

(10) $$\frac{1}{∆t}\propto f\left(v_{\mu }\right)+f\left(v_{p}\right)+f\left(v_{\varepsilon }\right)$$

With the rapid development of RPS technology, the core challenge faced by researchers is how to systematically elucidate the influence of different channel types on the signal generation mechanism in complex and variable detection environments. The three-way classification framework of biological, solid-state, and other inductive pores proposed in this paper aims to address this challenge. This classification is not a simple material science division but is based on the essential differences in pore formation principles, interfacial properties, and signal transduction mechanisms. Together, they form a complete knowledge chain from mechanism understanding to technology transformation, enabling researchers to accurately select or design appropriate pore systems in detection scenarios of different scales and needs, thereby systematically promoting the in-depth application of RPS technology in the fields of single-molecule detection, clinical diagnosis, and nano-medicine.

## 3. RPS Detection of Micro-Target

To accurately determine the size of the micro-target, it is crucial to evaluate the detected signal, i.e., the relationship between the measured magnitude of the signals, the geometry of the sensing gate, and the particle size. This relationship is normally based on measuring the resistance change induced by particle massing through the sensing orifice. With the development of micro-and nanofabrication technologies, different types of micro-and nano-scaled sensing channels have become available. This section focuses on the types of RPS sensing orifices, divided according to their properties and applications: (1) biological sensing pores, which aim to detect single molecules; (2) solid-state sensing pores, which are made of silicon-based materials like silicon nitride; and (3) other sensing pores, usually made of PDMS-based structures. In order to sensitively detect the targets using RPS, it is crucial to carefully select the sensing materials and optimize parameters such as the applied voltage and the volume ratio between the particle and the sensing gate.

### 3.1. Biological Sensing Pores

Based on their atomic-precision structural programmability, biological sensing pores are an ideal model system for revealing the fundamental physicochemical mechanisms of RPS. Its genetically engineered lumen charge distribution not only directly regulates the analyte capture efficiency but also accurately converts molecular structure information into quantifiable current-blocking signals through the instantaneous interaction between amino acid side chains and pores. This type of research provides a core paradigm for understanding the mapping relationship between molecular and signal features. Especially when the analyte scale is close to the critical size of the pore, the inherent structural specificity of biological sensing pores becomes a key factor in distinguishing conformational isomers. A summary of the relevant works is presented in Table 1.

**Figure 3:**
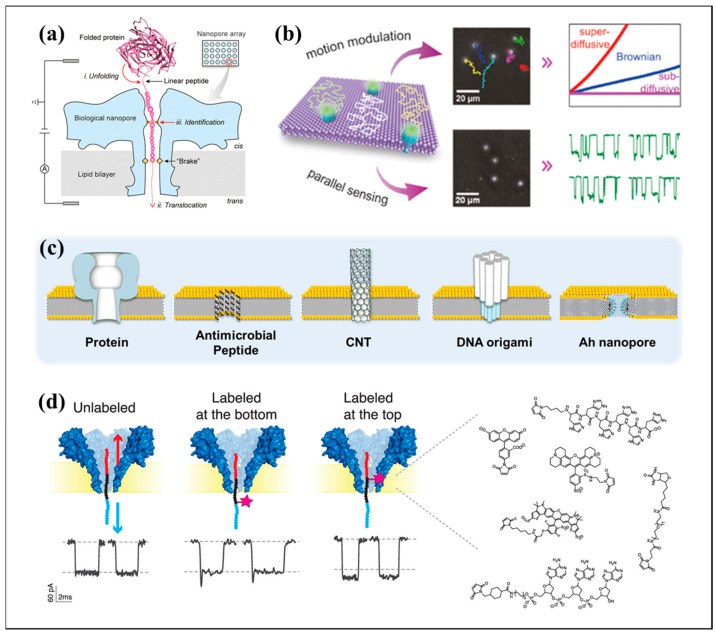
(**a**) Schematic representation of single-molecule protein sequencing using a biological nanopore. Reproduced from Ref. [43]. Copyright 2021, Wiley. (**b**) Imaging and tracking of α-HL nanopores in a DIB. Reproduced from Ref. [48]. Copyright 2018 ACS Publications. (**c**) Schematic representation of multiple types of nanopores formed in the lipid membrane. Reproduced from Ref. [51]. Copyright 2019 ACS Publications. (**d**) Investigation of labeled and unlabeled peptides via FraC. Reproduced from Ref. [56]. Copyright 2019 ACS Publications.

A significant advancement in this field is the application of biological pores for the detection of DNA, viruses, and antigens due to their high resolution and ease of use in single-molecule detection. This technique leverages the unfolding of structured proteins, controls peptide translocation, and identifies amino acids. As shown in Figure 3a, it highlights the potential of biological nanopores in protein sequencing and disease detection and discusses the challenges and future directions in the field [43]. Cao et al. demonstrated that aerolysin nanopores can sensitively detect poly D-adenine oligonucleotides of 2–10 nucleobases, with distinct current blockades and dwell times for different lengths. Their study reproduced and extended these findings using MECA technology, showing that poly d-cytosine oligonucleotides also produce resolvable current blockades, although with shorter dwell times and broader current peaks, suggesting the potential for distinguishing between different nucleotide species based on dwell time [44]. To further improve signal fidelity, a nanopore front-end readout integrated chip was designed for high-bandwidth and low-noise performance in biological nanopore sensing. The chip comprises a nanopore microelectrode chip and analog front-end chips, utilizes heterogeneous packaging technology and on-chip circuits to minimize parasitic capacitance, and features a 16-channel configuration. Each channel achieves a bandwidth surpassing 11 kHz and equivalent input noise below 3 pA, effectively detecting signals from target analytes passing through biological nanopores [45]. PlyAB toxin was engineered to form cylindrical nanopores with low electrical noise. They utilized continuum simulations to investigate the nanofluidic properties of these nanopores and discovered that engineering PlyAB’s inner surface charge could induce electro-osmotic vortices, facilitating the electrophoretic capture of large folded proteins [46]. A nanopore approach was then developed to analyze protein–DNA complexes. They used a hairpin DNA structure followed by ssDNA, allowing the distinction between DNA−protein complexes and bare DNA as they pass through a nanopore. This method provides equilibrium and kinetic information and can be used to test the inhibitory effects of small molecules on complex formation [47]. The fundamental applications and technical optimizations of biological sensing pores are focused on exploring the detection capabilities and enhanced sensing performance, laying the groundwork for their ability to detect diverse analytes with improved sensitivity and specificity. 

The potential of biological pores is further demonstrated by actively manipulating the pore environment to further improve the signal-to-noise ratio and throughput after confirming the recognition of multiple analytes. The corresponding key technological innovations are systematically described below. Then, a novel technique for modulating the motion of biological nanopores using osmosis-driven interactions is demonstrated to enhance optical single-channel recording (Figure 3b) for nucleic acid sensing. By adjusting the asymmetric salt concentrations across a droplet interface bilayer, they could control the lateral movement of nanopores and minimize thermal drift, thereby improving the signal-to-noise ratio [48]. Precise pore restriction engineering combined with signal amplification significantly enhances the performance of nanopore sensors. Their advancements in measurement throughput and spatial resolution promise to support both fundamental single-molecule biophysical research and practical precision-medicine applications [49]. 

Faced with the difficulty of translocating large molecular weight proteins, Zhang et al. demonstrated that attaching densely charged tags, such as DNA or peptide oligomers, to the termini of native proteins via chemical means can enhance their translocation through biological nanopores. They address the challenge of off-target modification of lysine side-chain primary amines in such tagging chemistry. By assaying native proteins from a variety of origins, they proved the universality of their approach, which can be generalized to diverse protein translocation architectures through nanopores. This advancement promises to boost nanopore applications in proteomics and make native proteins more accessible to researchers [50]. 

The use of alpha-hederin nanopores for discriminating single nucleotides is shown (Figure 3c). They found that Ah can spontaneously form nanopores in lipid membranes, and that these nanopores can be controlled in terms of size and formation rate. The Ah nanopores enabled the detection of single-stranded DNA homopolymers and the discrimination of four types of nucleotides based on the current blockades and dwell times [51]. Tag-based techniques have been proposed for protein translocation via nanopore sensors to enable single-molecule protein sequence analysis. They discuss how techniques like oligonucleotide conjugation and molecular motors can facilitate the movement of protein strands and peptides through protein nanopores. They also investigated site-specific protein conjugation chemistry as a prospective direction for single-molecule protein detection and sequencing of native proteins [52]. 

The sensing performance is enhanced through targeted technical adjustments, such as controlling nanopore motion, enhancing translocation efficiency, and expanding the detection scope. These efforts have contributed to overcoming side effects, including thermal drift, inefficient protein translocation, and limited nucleotide discrimination, extending the applicability of nanopores for complex analytes. With the maturity of experimental technology, researchers have begun to systematically explain the signal generation mechanism and application boundaries of biological pores from the perspective of computational simulations and system analyses. In the following section, the transition from the microscopic mechanism of action to the review of macroscopic research progress is completed by reviewing the representative literature. The physical mechanism underlying ionic current blockades in the biological nanopore MspA was clarified using all-atom molecular dynamics simulations. They found that the volume of water displaced from the nanopore by the DNA strand controls the ionic current, which is determined by the steric and base-stacking properties of DNA nucleotides. This leads to sequence-specific ionic current blockade [53]. Wang et al. summarized and discussed analytical strategies using biological nanopores, focusing on on-pore and off-pore strategies, and proposed future directions, including the discovery of new nanopores and optimization of analysis methodologies [54]. A comprehensive review of DNA sequencing technologies was provided, focusing on the evolution from first to third-generation sequencing methods. They highlighted the emergence of nanopore sequencing as a promising third-generation technology that enables direct, label-free, and real-time sequencing of single DNA molecules [55]. 

Focusing on the protein level, the use of biological nanopores has been explored for single-molecule protein fingerprinting (Figure 3d). Their study found that labeled and unlabeled peptides could be distinguished based on current blockades, with the most sensitive region for label detection identified near the pore constriction region. Labels with varying physicochemical properties produce distinct current blockades and translocation times, indicating the potential for using multiple labels in protein fingerprinting [56]. In order to break through the pore size limitation of α-hemolysin, large biological nanopores were utilized for single-protein detection by analyzing pore formation and protein translocation. They classified the current signals from five different pore-forming proteins and selected the perforin nanopore for its suitability in detecting granzyme B protein. This study demonstrated that granzyme B can penetrate perforin nanopores in an unfolded state, producing distinct current blockades. The authors proposed an analytical approach to identify appropriate nanopores for sensing applications and suggested that their method could be useful for exploring large biological nanopores beyond α-hemolysin [57]. 

A biological nanopore assembled from complement component 9 (C9) through self-assembly was developed for analyzing folded proteins at the single-molecule level. The poly (C9) nanopore enables the capture of analyte proteins by electro-osmotic flow, resulting in dwell times that exceed 300 μs. This research demonstrated the ability to ascertain the volumetric characteristics and configuration of untagged folded proteins with high precision. In addition, poly (C9) nanopores can differentiate between the open and closed conformers of adenylate kinase according to variations in current oscillations and shape modeling [58]. The combination of numerical simulations and experiments explains the current blockades, evaluates sequencing techniques, and develops methods for property characterization. Thus, the theoretical and practical understanding of biological sensing pores is deepened, which bridges fundamental biophysics with applied research and supports both basic science and potential clinical applications.

After systematically elucidating the mechanism of biological pore action and the model system, they focus on the direct detection of complex clinical samples and the expansion of their interdisciplinary applications, illustrating the transition of this technology from basic laboratory research to real-world clinical validation and translational applications. Subsequently, proteins at nanomolar concentrations in complex biological samples were detected using engineered biological nanopores. Inspired by the nuclear pore complex, the researchers integrated a film of structurally disordered polypeptides into the YaxAB nanopore to construct an entropic gate that substantially decreased the entry of non-target proteins. By introducing a specific recognition element within these polypeptides, the nanopore can selectively capture and identify target proteins directly from blood samples without prior preparation [59]. Jin et al. examined the utilization of nucleic acid-based biological nanopore sensing strategies in the detection of tumor markers, emphasized the importance of early cancer diagnosis and the role of tumor markers, highlighted the advantages of high sensitivity, specificity and label-free detection of biological nanopore technology, covering direct nucleic acid analysis detection and nucleic acid, biological nanopore-mediated indirect detection, involving a variety of tumor marker detection, and discussed the challenges of low throughput and membrane stability and proposed future research directions [60]. The application of biological nanopore technology for monitoring chemical changes at the molecular level is then reviewed. They summarized the use of nanopores for detecting various chemical processes, including enzymatic reactions, self-assembly, photoisomerization, and the formation and cleavage of covalent bonds. Their study highlights the potential of nanopore technology for real-time, single-molecule detection and analysis of chemical reactions, suggesting its broad application in both biological and chemical research [61]. 

Biological sensing pores have emerged as a vital method for single-molecule detection, driving the transition of biological sensing pores from laboratory research to practical use, with applications ranging from targeted protein detection in blood to early cancer diagnosis and real-time chemical reaction monitoring. Ongoing efforts to refine biological sensing methods underscore their potential in precision medicine, diagnostics, and chemical research, while acknowledging areas for further improvement. 

**Table 1:** Summary of research on RPS in biological sensing pores.

Serial No.	Micro/Nano-Target		Electric Field Signal		Sensing Pore Information	Ref.
	Type	Material	Voltage	Frequency	Type	
1	Molecules	Poly4	100 mV	11 kHz	Wild-type aerolysin	[45]
2	Molecules	Cytolysin A	200 mV	10 kHz	Cytolysin A	[62]
3	Molecules	ssDNA	200 mV	10 kHz	Alpha-hederin	[51]
4	Molecules	granzyme B	200 mV	20 kHz	Perforin	[57]
5	Molecules	DNA/RNA	120 mV	50 kHz	Wild-type α-hemolysin	[63]
6	Proteins	Protein biomarkers	400 mV	100 kHz	Wild-type aerolysin	[64]
7	Molecules	Peptide	90 mV	500 kHz	Fragaceatoxin C	[56]
8	Molecules	DNA Sequence	180 mV	100 kHz	MspA	[53]
9	Molecules	polydeoxyadenines	120 mV	10 kHz	Aerolysin	[65]
10	Molecules	DNA hybrids	120–160 mV	100 kHz	DPhPC bilayer	[66]
11	Molecules	α-syn124–140	100 mV	10 kHz/200 kHz	Toxin aerolysin	[67]
12	Cells	Ramos cells	80–120 mV	10 kHz	Aerolysin	[68]
13	Proteins	Plasma Proteins	50/100 mV	10 kHz	Pleurotolysin toxin	[46]
14	Proteins	nucleocapsid protein	100–120 mV	100 kHz	α-hemolysin	[69]
15	Proteins	Nanomolar proteins	75 mV/150 mV	50 kHz	YaxAB	[59]
16	Molecules	DNA Complexes	100 mV/120 mV	20 kHz	α-hemolysin	[47]
17	Proteins	Folded proteins	150 mV	20 kHz	Poly (C9)	[58]
18	Molecules	miRNA	150 mV	50 kHz	α-hemolysin	[70]
19	Proteins	Streptavidin	35–100 mV	0.1 Hz–1 MHz	Lipid bilayers	[71]
20	Molecules	prion protein	100 mV	10 kHz	DNA aptamer	[72]

### 3.2. Solid-State Sensing Pores

Solid-state sensing pores exhibit good material stability and scalability. Due to their excellent material stability and microfabrication compatibility, they serve as novel models to promote techniques from mechanism verification to a new stage of application expansion, including silicon-based materials and two-dimensional materials. The precise and adjustable pore size and flexible surface chemical modification capabilities of RPS technology enable it to overcome the inherent size limitations of biological pores and to be successfully applied to the detection of complex systems, such as ribosomal complexes and metal-organic cage-labeled small molecules. More importantly, the inherent integrability of solid-state channels provides an ideal platform for constructing a signal amplification-target capture modular system, thus revealing the engineered implementation path of RPS technology to achieve highly sensitive detection in complex biological samples. The relevant research and corresponding key details are presented in Table 2. 

**Figure 4:**
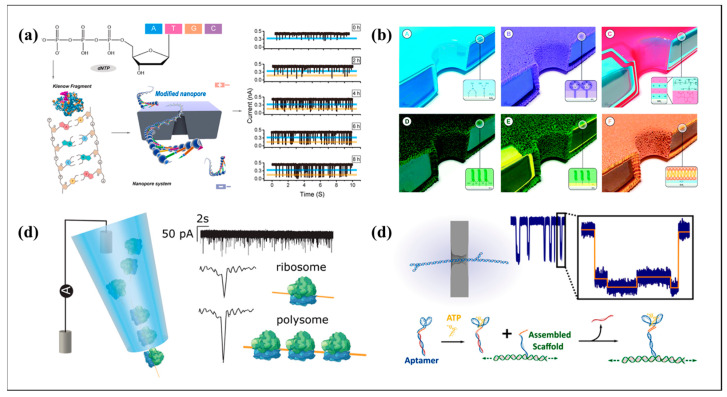
(**a**) Schematic representation of DNA synthesis via synthetase and current signal detection from synthetase-based DNA synthesis. Reproduce from Ref. [73]. Copyright 2025 Elsevier. (**b**) Schematic illustration of the idealized cartoon renderings of the applied nanopore coatings. Reproduce from Ref. [76]. Copyright 2019 Royal Society of Chemistry. (**c**) Schematic of the nanopipette sensing configuration and typical ion current profile. Reproduce from Ref. [79]. Copyright 2019, ACS Publications. (**d**) Schematic illustration of short DNA scaffolds for structural identification using solid-state nanopores. Reproduce from Ref. [81]. Copyright 2017, ACS Publications.

The chemically modified solid-state nanopores achieved long-term stability for several days, allowing for dynamic monitoring of DNA synthesis processes (Figure 4a). The modified nanopores enabled the monitoring of DNA synthesis by polymerase for over 10 h, revealing synthesis rates of approximately 1.33–1.78 kb/min. This research highlights the potential of long-lived solid-state nanopores for biochemical detection and other applications [73]. A fabrication method for high-precision nanopores was developed to improve the spatial and temporal resolution. Additionally, they discussed methods for slowing down biomolecule translocation to improve the temporal resolution. These advancements aim to optimize solid-state nanopore DNA sequencing for single-nucleotide recognition and single-biomolecule detection applications [74]. By reviewing the applications of transmission electron microscopy (TEM) in solid-state nanopore technology, from fabrication to bio-imaging, they highlighted the use of TEM for precise nanopore drilling and tuning, as well as its advantages over other fabrication methods. It also covers the imaging of nanopore geometries using 3D reconstruction techniques and the challenges and methods for imaging DNA origami structures. Additionally, the authors proposed the integration of liquid TEM with solid-state nanopores for in situ bio-imaging, suggesting a new direction for the real-time observation of molecular translocation dynamics [75]. 

When the pore geometry approaches the physical limit, the dimension of surface chemical functionalization emerges, such as the synergistic breakthroughs made by coating engineering in the field of ion and small-molecule detection. The application of coatings, such as vapor deposition, surfactants, physical adsorption, and layer-by-layer self-assembly in solid-state nanopores, is summarized (Figure 4b). Comparisons were conducted regarding the coating preparation method, coating stability, and requirements for special devices in its preparation. This has promoted the development of hybrid bio-synthetic nanopores and their extension to the detection of non-aqueous environments [76]. The evolution of solid-state nanopores for analyzing ions and small molecules is demonstrated, focusing on fabrication methods, ion transport mechanisms, and applications in small-molecule detection. They discussed various fabrication techniques, such as ion-beam sculpting, e-beam lithography, track-etching, controlled dielectric breakdown, and nanopipette pulling. The review also covered ion transport phenomena, including ionic current rectification and ion selectivity, and highlighted the potential of solid-state nanopores in bioanalysis and energy applications [77]. 

An innovative detection approach was developed for carcinoembryonic antigen (CEA) using a solid-state nanopore. This technique capitalizes on the specific interactions between aptamer-functionalized magnetic Fe_3_O_4_ nanoparticles and CEA. The aptamers are hybridized with tetrahedral DNA nanostructures (TDNs), which are released upon CEA binding. The passage of TDNs through the nanopore induces discernible current blockades, facilitating the sensitive detection of CEA down to 0.1 nM [78]. Solid-state nanopore technology efficiently distinguished 80S ribosomes and polysomal complexes in human neuronal cell lines and Drosophila ovarian samples (Figure 4c). The results showed significant differences in the amplitude and residence time of the two ribosomes, which could be adopted as a basis to discriminate between the two types of complexes in the sample mixture. This research underscores the capability of solid-state nanopores for label-free, rapid detection and characterization of ribosomal complexes [79]. 

Currently, the key challenges are the difficulties in fabricating ultra-small nanopores and ultra-thin membranes, controlling DNA translocation speed, and detecting base-specific signals. By developing a bidirectional DNA motion control system, single-nucleotide discrimination in DNA molecules was enabled, showing distinct current blockades and dwell times for different nucleotides [80]. The stability and scalability of solid-state sensing pores are leveraged, which focuses on core optimizations and biomolecular detection, including chemical modification for long-term stability, fabrication advances, and improved resolution.

Based on the longitudinal deepening of materials development, coating engineering, and sequencing applications, the research frontier has shifted to a new paradigm of structure-mediated indirect sensing and horizontal integration of microfluidics-3D printing. The following system illustrates the system construction strategy of the solid-state sensing aperture to realize modular function expansion in complex scenarios. Subsequently, a DNA origami-based approach was presented to identify molecular substructures along nucleic acid sequences using solid-state nanopores. Their method involves the creation of DNA scaffolds with specific protrusions and detecting these structures electrically (Figure 4d). This study demonstrated that solid-state nanopores can differentiate DNA scaffolds with zero, one, and two dsDNA protrusions. They also showed that small molecules like ATP, which are too small to be detected directly by conventional nanopore methods, can be sensed indirectly by triggering structural changes in the DNA scaffold [81]. The dissociation and passage of DNA duplexes via a sub-2 nm silicon nitride nanopore were demonstrated, producing three-level blockades. They utilized a simple model to explain the unzipping and translocation processes. The current patterns generated are crucial for determining and quantifying analytes using solid-state nanopores [82]. Li et al. demonstrated a simple and cost-effective technique for fabricating hourglass-shaped solid-state nanopores and nano trenches using plasma-enhanced chemical vapor deposition (PECVD). They achieved nanopores with dimensions as small as 19 nm × 45 nm and nano trenches as narrow as 7 nm. The hourglass-like morphology of the nanopores, characterized by a narrow waist, was attributed to the step coverage feature of PECVD. This method allows the mass production of uniform nanopore arrays, which have potential applications in the parallel analysis of biomolecules [83]. 

A cost-effective and reusable microfluidic device was developed to integrate solid-state nanopores using 3D-printing technology. The device allows for rapid analyte changes and reversible chip integration, thereby reducing the risk of cross-contamination. They investigated ionic transport through nanopores under various conditions and demonstrated their use for sensing polymer conformations and protein–urea interactions at the nanoscale [84]. The activity of protein–DNA complexes bound to nanopores was elucidated by tracking ionic current blockades. They found that monovalent streptavidin–DNA complexes produced fluctuating current blockades at distinct levels, attributed to the movement of the protein plug and its interactions with the nanopore surface [85]. Innovative solid-state pore strategies, including DNA origami-based sensing for small molecules, DNA duplex analysis, cost-effective fabrication, 3D-printed microfluidic integration, and protein–DNA complex dynamics, are featured, enhancing detection capabilities and practicality.

By combining the research progress of the entire chain of solid-state sensing channel preparation, functionalization, integration, and application, the core roadmap of its future development is summarized below, focusing on ultra-high spatiotemporal resolution sensing, high-specificity molecular recognition, and epigenetic analysis. The fabrication methods of solid-state nanopores and the progress in DNA single-molecule sequencing were summarized, which provided methods to improve spatial and temporal resolution, pointed out new detection methods such as tunneling current sensing and multi-channel sensing, and finally summarized the development and prospects of nanopore technology in the future [86]. Bio-inspired solid-state nanopores were designed featuring a thin, narrow constriction as the sensing region, inspired by successful protein nanopores used in DNA sequencing. Bio-inspired nanopores can achieve a high spatial resolution comparable to that of 2D material nanopores while markedly decreasing the noise level [87]. A comprehensive perspective was provided on boosting the capability of selecting solid-state nanopore sensing techniques through pore-in modification strategies. They summarized various modification methods, including small-molecule functionalization, metal coordination, nucleic acid hybridization, peptide/protein interactions, and pore-in-pore structures. These modifications enable the selective detection of ions, peptides, small molecules, and proteins by mimicking the selectivity filters of biological ion channels [88]. Rapid and specific quantification of 5-hydroxymethylcytosine (5 hm C) in mammalian genomic DNA was achieved using a solid-state nanopore assay. The assay uses a biotinylated DNA probe selectively functionalized at 5 hm C sites via enzymatic labeling and subsequent click chemistry. When these labeled DNA fragments bind to a monovalent streptavidin protein, they form a nucleoprotein complex that produces resolvable current blockades when translocated through a solid-state nanopore [89]. 

Solid-state sensing pores have emerged as powerful tools for the detection of complex systems, enabling researchers to overcome the inherent size limitations of biological pores and improve existing techniques. The inherent integrability of solid-state sensing pores has led to breakthroughs, overcoming previous sensitivities, and yielding unexpected results. In addition, novel interdisciplinary construction of unique systems has enhanced the signal amplification of targeted particles. These unique advantages enable the detection of complex biological samples with high resolution. Advancements in solid-state sensing methods have been synthesized for fabrication and sequencing, bio-inspired narrow-constriction designs, modification strategies for selectivity, and detection of specific biomarkers, bridging technical refinement and real-world diagnostics.

**Table 2:** Summary of research on RPS of solid-state sensing pores.

Serial No.	Micro/Nano-Target			Electric Field Signal		Sensing Pore Information		Ref.
	Type	Size (Diameter/Length)	Material	Voltage	Frequency	Type	Size (Diameter/Width/Height)	
1	Proteins	—	Carcinoembryonic antigen	80–120 mV	100 kHz	SiNx	4 nm	[78]
2	Molecules	—	dsDNA.	100 mV	500 kHz	SiN	3 nm	[81]
3	Molecules	—	DNA	200 mV	250 kHz	SiN	8.5 nm	[90]
4	NPs	80 nm/100 nm	PS	100 mV	30 kHz	SiN	200 nm	[91]
5	Cells	—	human PANC-1/HPNE cells	1–2 V	3, 10 kHz	Quartz capillaries	30 nm	[92]
6	Virus	—	HBV	10 V	1 kHz	Glass	50 nm/50 nm	[93]
7	NPs	160 nm	PMMA	200 mV	1 MHz	Borosilicate glass capillaries	340 nm	[94]
8	NPs	50 nm/ 100 nm	PS	500 mV	3.2–10 GHz	Si_3_N_4_	450 nm	[95]
9	NPs	100 nm	PS/*E. coli*	1.2 V	10 kHz	Glass	150 nm	[96]
10	Virus	—	Adeno-associated virus	100–300 mV	10 kHz	SiN	66 nm/90 nm	[97]
11	NPs	70 nm	Latex nanoparticles	200 mV	7.3 kHz	Quartz capillaries	—	[98]
12	Molecules	—	Enzyme molecules	500 mV	100 kHz	Pt	250 nm	[99]
13	Bio-pellets	60–160 nm	EV	1 V	100 kHz	D263 glass	200 nm	[100]
14	Bio-pellets	60 nm/90 nm	EV	400 mV	10 kHz	Gold	—	[101]
15	Virus	30–35 nm	HBV	0.5–2.5 V	10 kHz	D263 glass	60 nm/60 nm	[102]
16	Proteins	—	Aβ42	500 mV	20 kHz	Quartz nanopipette	34 nm	[103]
17	Bio-pellets	—	ADSC-EVs	320 mV	10 kHz	SiN	—	[104]
18	Molecules	—	miRNA	1–5 V	1 kHz	Gold	—	[105]

### 3.3. Other Sensing Pores

Other sensing pores, such as PDMS-based nanopores, have overcome the key bottlenecks of traditional RPS in terms of detection throughput, cost control, and scenario adaptability through the integration of material system innovation and microfluidic technology. PDMS-based nanopores are created via track-etching and offer cost and reproducibility benefits. They can be customized for specific applications through surface modifications, like PEGylation, and use capillary force to complete electrolyte filling independently, without relying on external instruments, which significantly promotes RPS technology from the laboratory environment to on-site real-time detection. Dual-channel time-of-flight analysis technology can achieve efficient identification of single peptides at millisecond-level temporal resolution through the synergistic integration of electrophoretic separation and RPS detection. Such research bridges the traditional boundaries between sensing channels and microfluidic functional components and drives the paradigm of RPS technology to transition from single-particle counting to multi-dimensional physical parameter analysis. The relevant research and corresponding key parameters are listed in Table 3. 

The pore diameter and shape were well controlled by milling the lamellae and drilling through circular nanopores in glass using FIB milling. These nanopores enhanced the detection of HBV capsids via resistive pulse measurements, showing a fivefold increase in pulse amplitude relative to that of rectangular pores. Serial nanopores improved the resolution between T = 3 and 4 capsids by up to two times. Pores near the capsid size (<45 nm) showed a tighter fit [106]. Following the optimization of the geometric topography, a silver nanoneedle probe with an in situ AgCl layer was created to address the current decay in nanopore sensing. Unlike gold nanoneedles, the Ag/AgCl interface eliminates double-layer charging, maintaining stable DC currents for α-hemolysin nanopore. The platform detected sulfonated β-cyclodextrin binding events with a linear frequency-concentration correlation and 81.2% current blockades [107]. Vogel et al. advanced the adjustable measurement of the diameter of the zeta potential of individual nanoparticles simultaneously by integrating convection into the electrokinetic model. Using voltage and pressure modulation, they tracked particle translocation through conical pores, enabling robust single-particle analyses of complex samples. This TRPS approach facilitates precise nano-bio interaction studies and meets the regulatory demands for particle-by-particle characterization in nano-medicine [108]. In addition, ligand/zwitterion hybrid-functionalized gold nanoparticles can specifically detect the influenza A H1N1 virus using resistive pulse sensing. The nanoparticles combine a sialic acid receptor specific to human influenza with a zwitterionic layer to reduce non-specific binding. When exposed to H1N1, these nanoparticles bind to viral hemagglutinin, causing a 20–40 nm shift in the RPS-measured size distribution. Control nanoparticles lacking the receptor or zwitterion layer showed no significant shift [109]. Material-modified sensing pores have been developed for biological and physical applications. Surface modification is necessary to enhance the sensitivity and stability of the sensor.

After the technical foreshadowing of the pore structure and probe interface, the focus of the research quickly shifted to high-fidelity quantitative analysis of complex biological samples. In the following, typical cases such as standardized EV detection, DNA-peptide hybrid vectors, time-of-flight analysis of dual nanopores, and electro-osmotic directional transport systematically demonstrate the wide adaptability of RPS technology from laboratory research to clinical/field application scenarios. Microfluidic resistive pulse sensing was used to standardize the measurement of extracellular vesicles and sub-micro-scaled targets in biofluids. Reproducible size distributions were achieved at specific dilutions for the different biofluids. Using overlapping MRPS cartridges, they extended the dynamic range to 65 nm–2 μm with a high concordance. This study provides validated guidelines for robust EV/particle quantification, addressing sensitivity and reproducibility issues in diagnostic applications [110]. A hybrid DNA-peptide nanocarrier was created for biomarker detection using RPS. They functionalized superparamagnetic beads with CRP-binding peptides and non-binding DNA oligomers. Hybrid carriers enhanced the signal compared to pure peptides, with optimal performance using 10-base DNA. This method is label-free, specific, and expands the applicability of RPS to peptide libraries [111]. 

**Figure 5:**
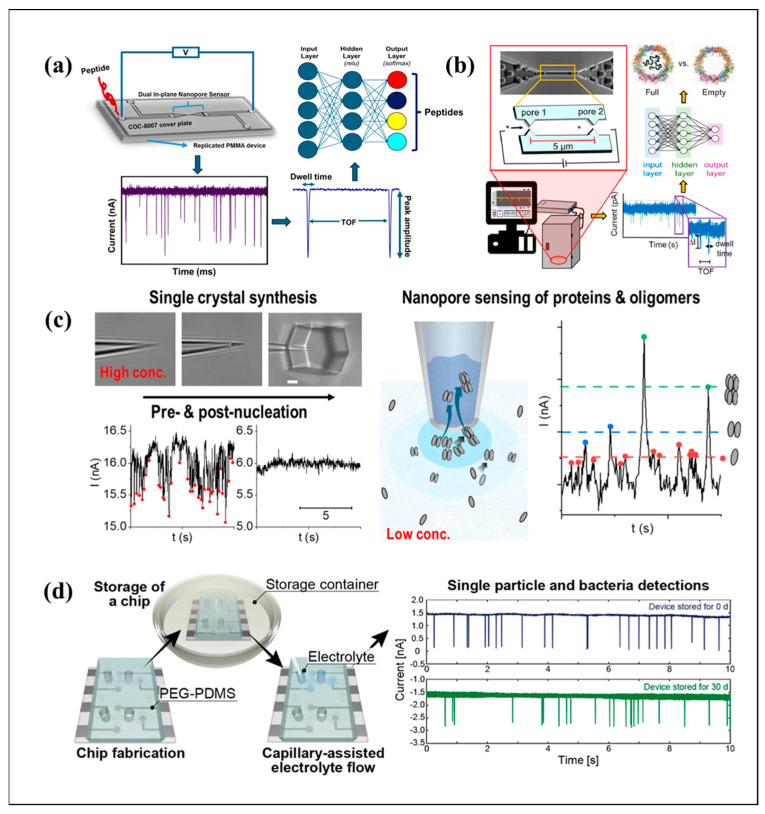
(**a**) Electrokinetic transport of metenkephalin through PMMA/COC dual in-plane nanopore sensors and classification of peptides with machine learning via a neural network. Reproduce from Ref. [112]. Copyright 2024 ACS Publications. (**b**) Experimental setup and SEM images of the Si master, and classification with supervised ML via a neural network. Reproduce from Ref. [113]. Copyright 2024 Elsevier. (**c**) Controlled nucleation and crystal growth of lysozyme in D_2_O and H_2_O, experimental setup, and mass transport directions. Reproduce from Ref. [117]. Copyright 2025 Elsevier. (**d**) Capillary-assisted electrolyte flow in a PEG–PDMS microfluidic chip stored over time and single-particle detection using stored chips. Reproduce from Ref. [120]. Copyright 2023 ACS Publications.

To overcome the limit of single-hole resolution, Chibuike et al. achieved millisecond-scale, label-free single-peptide identification using a thermoplastic dual-nanopore sensor integrated with nanoscale electrochromatography (Figure 5a). The PMMA/COC device included a 5 μm-long nanochannel flanked by two nanopores. Peptides were identified based on the time-of-flight, normalized current amplitude, and dwell time from both pores [112]. Furthermore, salt concentration gradients and AC fields can induce unidirectional electro-osmotic flow in ultra-thin nanopores, regardless of the surface charge. This flow is linearly controlled by the bulk salt concentration bias in sub-10-nm pores and allows continuous nanoparticle pumping under AC fields. They developed an AC resistive pulse-sensing prototype using a monolayer MoS_2_ nanopore, where nanoparticles consistently moved toward the low-concentration reservoir, generating measurable current blockades [38]. 

After the successful introduction of microfluidic integration, electroosmosis manipulation, and machine learning into RPS technology, noise suppression, artifact recognition, and data intelligence have become the core topics of the next stage of research. A comprehensive upgrade of the technology from noise reduction optimization at the physical level to intelligent empowerment at the algorithm level is involved, focusing on innovative research such as tunable channel design, particle trajectory error analysis, and machine learning-based viral typing. Innovations in resistive pulse sensing for microfluidic bioanalysis include tunable pores, multi-electrode designs, and noise reduction. Tunable pores via elastic membranes or hydrodynamic focusing dynamically adjust the constriction size, thereby expanding the particle sizing range. Multi-electrode architectures generate complex pulse signatures to reduce false positives and improve the selectivity [39]. 

The pulse size distributed in TRPS broadens due to off-axis particle trajectories, especially in larger pores or under higher pressure-driven flow. Using monodisperse polystyrene nanoparticles (200−800 nm), they observed bimodal distributions, with particles near the pore edge generating larger pulses due to electric field enhancement. Pressure reversal experiments confirmed that trajectory variation, not particle dispersity, caused broadening, with CVs of 4.4% for 400 nm particles and 1.0% for 800 nm particles [25]. Nguyen et al. created a dual in-plane nanopore sensor in thermoplastic substrates through nano-injection molding to differentiate full and empty rAAV capsids at the single-particle level using RPS and nanoscale electrophoresis (Figure 5b). The sensor identifies capsids based on differences in their surface charge and internal cargo. Machine learning achieved 98.5%accuracy via PCA and K-means clustering, with neural networks further improving the results [113]. In viral typing applications, a PD-functionalized nanopore with RPS was developed to specifically detect H1N1 influenza A virus particles. Virus-specific ligands were immobilized on a polyurethane pore surface using PD, increasing the virus translocation time from 0.28 to 1.74 ms. Characteristic pulse shapes with transient-current pauses were observed during virus trapping. Machine learning distinguished specific and non-specific surfaces with 83.5% accuracy, showcasing its potential for stochastic binding analysis in biosensing [114]. The RPS assay rapidly quantifies site-specific DNA methylation in genomic targets without bisulfite conversion or sequencing. They used streptavidin-coated superparamagnetic beads functionalized with biotinylated DNA probes to capture the target sequences. Anti-5-methylcytosine antibody binding to methylation sites alters the bead surface charge, reducing the nanopore translocation velocity [115]. Innovations in RPS for bioanalysis are highlighted, with a focus on standardized measurements, multi-pore designs, field control, and specific biomolecule detection.

Based on signal amplification, noise suppression, and machine learning algorithm technology systems in place, low-abundance small-molecule detection, dynamic protein aggregation process analysis, and on-site rapid detection have become the final three challenges in testing the performance of RPS technology in extreme application scenarios. The deployment of improved sensitivity and on-site rapid detection platforms has emerged from cutting-edge studies. The lanthanide-based metal-organic cage (PCC-57) amplified nanopore signals for sub-4 kDa biomolecules via phosphate-chelating interactions. Using oligonucleotide linkers, PCC-57 enabled the detection of angiopep-2 peptides (2.4 kDa) and polyphosphoric acid, with distinct current blockades and dwell times. Functionalization of PCC-57 with thrombin-binding aptamers allowed the specific detection of human α-thrombin in a physiological buffer, with ΔI/I_0_ ≈ 0.01 and voltage-dependent translocation times. This approach has the potential for clinical biomarker screening [116]. 

Resistive pulse sensing with single nanopipettes enabled the real-time detection of pre-nucleation activities during lysozyme crystallization in D_2_O, with transient current spikes resolving dynamic protein oligomer translocation and aggregation at the single-entity level (Figure 5c). Electrochemically controlled localized supersaturation was employed to capture current signatures dependent on applied potential and protein concentration, revealing both oligomer translocation through the nanopore and their transformation into larger aggregates prior to irreversible nucleation [117]. 

In high-noise environments, a deep learning method was developed to denoise ionic current signals in solid-state nanopores, enabling high-resolution tracking of single-nanoparticle translocation dynamics. When applied to a nano-corrugated SiO_2_ nanopore, the method revealed obscured features in the resistive pulses of 182 nm polystyrene nanoparticles, including five distinct current dips corresponding to nanoparticle passage through protrusions and entrance/exit signatures. The optimized AutoEnc04 neural network outperformed the conventional filters without temporal resolution loss. This label-free denoising approach facilitates real-time nanoscale motion analysis of biomolecules in noisy environments [118]. DNA zyme-based RPS sensor was then created for Ca^2+^ detection by monitoring DNA structural changes on the nanoparticles. Ca^2+^ binding induced DNA zyme cleavage and rearrangement, altering nanoparticle translocation speeds with a biphasic response: an initial speed decrease followed by an increase to 1.23 ms^−1^. The assay achieved a 1 μM LOD in 30 min, tunable to 0.3 μM with 90 min of incubation [119]. A capillary-assisted microfluidic RPS platform was developed using PEGylated PDMS chips for on-site single-particle analysis (Figure 5d). The one-step PEG modification maintained surface hydrophilicity for 30 days, enabling spontaneous electrolyte filling via capillary action without external instruments. The platform detected and sized 1–2 μm polystyrene particles and *E. coli* cells, resolving mixtures with distinct pulse heights [120].

Through their diverse capabilities and applications, other sensing pores showcase their potential for creating PDMS-based structures with customizable geometries, making them ideal for various advanced detection applications. For enhanced research and broader perspectives, further studies on the effectiveness of RPS combined with intelligent sensing systems are encouraged to serve as a bottom-up method for complex sensing samples.

**Table 3:** Summary of research on RPS of other sensing pores.

Serial No.	Micro/Nano-Target			Electric Field Signal		Sensing Pore Information		Ref.
	Type	Size (Diameter/Length)	Material	Voltage	Frequency	Type	Size (Width/Height)	
1	Bio-pellets	30–100 nm	EV	1–5 V	1–5 kHz	PDMS	—	[121]
2	NPs	100 nm/300 nm/350 nm	PS	0.4–4 V	5–50 kHz	PMMA	—	[122]
3	Molecules	—	λ-DNA	1 V	—	PMMA-COC	154 nm/203 nm	[123]
4	Molecules	—	ssDNA	5–20 V	10 kHz	PET	—	[124]
5	NPs	100 nm	PS	10 V	500 kHz	PDMS	—	[125]
6	NPs	160 nm	Nanoparticle	200 mV	10 kHz	—	250 nm	[119]
7	Virus	—	SARS-CoV-2	0.1–1 V	100 kHz	PDMS	—	[126]
8	Bio-pellets	30–200 nm	EV	10 V	10 kHz	—	—	[127]
9	NPs	15 μm/20 μm	PS	1–5 V	1–10 MHz	PDMS	40 μm/35 μm	[128]
10	Molecules	—	100 bp DNA	600 mV	5 kHz	PET	21 nm/23 nm/27 nm/30 nm/42 nm	[129]
11	Virus	68–77 nm	CNP	500 mV	1 GHz	SiN	—	[130]
12	Virus	129–141 nm	VLP	200 mV	—	NP200	220 nm	[131]
13	Proteins	30–150 nm	miR-21-5p	—	—	NP100	—	[132]
14	NPs	700 nm/830 nm	PS	1 V	2 kHz	PDMS	—	[133]
15	NPs	300 nm/1 μm	PS	4 V	100 kHz	PDMS	—	[134]
16	Proteins	75 nm	PC3 CM	5 V	100 kHz	Ts-400	375 nm	[110]
17	NPs	600 nm/1 μm	PS/ Yeast cells	10 V	400 kHz	PDMS	2 μm	[135]
18	Bio-pellets	252 nm/460 nm	EV	10–20 V	400 kHz	PDMS	500 nm/600 nm	[136]
19	Bio-pellets	50–330 nm	EV	700 V	100 kHz	—	47 nm	[137]
20	Molecules	200 nm	MDA-MB-231/PS	10–25 V	200 kHz	PDMS	10 μm/10 μm	[138]

## 4. Advantages, Challenges, and Future Scope

This work reviews the advantages of RPS platforms based on biological sensing pores, solid-state sensing pores, and other sensing pores. The hallmark capability of the analytical system with RPS technology as the core lies in the high-precision characterization of increasingly small particles, ions, and nucleotides, and is closely integrated with current cutting-edge trends, aiming to provide research orientation for high-potential applications. Through the collaborative innovation of nanofabrication and microfluidic integration, RPS sensors have achieved high-sensitivity and high-throughput detection and are widely used in biomedical research, clinical diagnosis, and environmental monitoring with their strong single-particle resolution capabilities, adaptability of multi-pore structure, and miniaturized integration.

Furthermore, for practical implementation, it is essential to detect complex samples with improved sensitivity and high throughput. Attempts have already been initiated to address these bottlenecks, where the major challenges are the breakthrough of the inadequacy of a single indicator to a systemic sensing platform. When the size of the target is close to the sub-nanometer scale or molecular weight, the multiple superposition effects of thermal, dielectric, and amplifier noise result in an unexpected and limited signal-to-noise ratio. Especially when coupled with complex sample interference, high ionic strength, protein adsorption, and particle/molecule heterogeneity in complex matrices such as serum, saliva, or environmental water samples, can significantly induce non-specific blocking and particle trajectory shifts, resulting in a significant increase in the false positive rate. In the case of solid-state channels, a machining error at the sub-2 nm level can cause an effective dimensional drift. However, the operating life of lipid membrane bio-pores is often shorter than 30 minutes at 37 °C and high shear stress. Neither of these types of orifices can meet the demanding requirements for point-of-care or field environments in terms of long-term equipment stability. Hence, field consistency is always a significant barrier.

To overcome the existing bottlenecks, future research should focus on developing bio-solid-state fusion channels, i.e., an innovative structure with genetically engineered protein rings as functional linings and two-dimensional materials as support frameworks, which should be used to simultaneously achieve sub-nanometer resolution and long-term stability. The integration of DNA origami structures or metal-organic cages within the pore channel to implement an in situ signal amplification strategy for a target small molecule ultimately achieves ultra-high sensitivity detection. In addition, based on the intelligent noise analysis model, the original current noise signal, particle motion trajectory, and potential molecular structure parameters are synchronously inputted, where real-time noise suppression, accurate trajectory correction, and automatic identification and annotation of molecular fingerprints can be accurately achieved. Finally, RPS technology is expected to become a universal technology platform that seamlessly connects basic scientific research, precision medicine practices, and on-site rapid detection needs. The commercial applications of RPS techniques are gaining traction, with key industry players driving innovation and expanding their adoption. As RPS evolves from research to commercialization, partnerships between biotech firms, tech giants, and diagnostic companies will accelerate its adoption in healthcare, environmental monitoring, and precision medicine.

## Data Availability

Not applicable.
